# Comparison of outcomes between cortical screws and traditional pedicle screws for lumbar interbody fusion: a systematic review and meta-analysis

**DOI:** 10.1186/s13018-019-1311-x

**Published:** 2019-08-23

**Authors:** Tingxin Zhang, Nana Guo, Tiantian Chen, Jinglong Yan, Wei Zhao, Gongping Xu

**Affiliations:** 1 Department of Orthopedics, The Second Affiliated Hospital of Harbin Medical University, 148 Baojian Road, Harbin, 150081 China; 2 Department of Critical Care Medicine, Harbin Medical University Cancer Hospital, Harbin, China

**Keywords:** Pedicle screw, Cortical screw, Cortical bone trajectory, Lumbar fusion, Meta-analysis, Systematic review

## Abstract

**Purpose:**

The clinical outcomes of using a cortical screw (CS) for lumbar interbody fusion were evaluated by comparison with conventional pedicle screw (PS) fixation.

**Methods:**

All of the comparative studies published in the PubMed, Cochrane Library, MEDLINE, Web of Science, and EMBASE databases recently as 18 March 2019, were included. All outcomes were analyzed by using Review Manager 5.3.

**Results:**

Twelve studies were included with a total of 835 patients, and two of the studies were randomized controlled trials. The outcomes of the meta-analysis indicated that the use of CS fixation for lumbar interbody fusion was better than conventional PS fixation in regard to operating time (*p* = 0.02), intraoperative blood loss (*p* < 0.00001), length of stay (*p* = 0.02), incidence of complications (*p* = 0.02), adjacent segmental disease (ASD) incidence (*p* = 0.03), and Oswestry Disability Index (ODI) (*p* = 0.03). However, there were no statistically significant differences in the back and leg pain visual analog scale (VAS), Japanese Orthopaedic Association (JOA) scale, and intervertebral fusion rate (all *p* > 0.05) between the CS fixation group and the PS fixation group.

**Conclusions:**

Based on this systematic review and meta-analysis, our outcomes indicated that both CS and conventional PS can result in good postoperative outcomes in lumbar interbody fusion. No significant differences were found in the back and leg pain VAS, JOA scale, and intervertebral fusion rate. However, CS fixation is superior to PS fixation in the following measures: operating time, intraoperative blood loss, length of stay, incidence of complications, ASD incidence, and ODI.

**Trial registration:**

PROSPERO registration number is CRD 42019132226.

## Introduction

Lumbar interbody fusion is a safe and effective treatment for lumbar degenerative diseases which can cause unstable segments [1–3]. Currently, pedicle screw (PS) is mainly used for internal spinal fixation. Moreover, PS internal fixation technology is widely used in a variety of diseases, such as spondylolisthesis, fracture, spinal deformity, tumor, and lumbar degenerative diseases, and has resulted in good postoperative outcomes. This technique is considered the gold standard for spinal fixation [4]. However, for patients with weakened bones, the PS pull-out strength is reduced due to osteoporosis, which can further lead to the loosening of the screw [5]. Studies have shown that non-osteoporosis patients have a screw loosening rate between 1% and 15%, and osteoporosis patients have a screw loosening rate greater than 60% [6]. In addition, the traditional PS is placed from the lateral to the medial trajectory [7]. Extensive tissue exposure is required when inserting a PS, resulting in longer surgical incisions, large intraoperative blood loss, and excessive muscle and soft tissue damage [8].

To overcome these troubles, Santoni et al. [9] first reported cortical bone trajectory (CBT) screw technology in 2009, which can improve the pull-out strength of screws in osteoporotic bones. The CBT screw was reported to have 1.71 times higher insertion torque than the traditional PS in vivo [10]. Many biomechanical studies have demonstrated that the CBT screw has good mechanical properties in lumbar spine specimens [11, 12]. Moreover, the cortical screw (CS) is placed from the medial to the lateral trajectory. It can effectively reduce the invasion rate of facet joints, the damage to muscle tissues, and the risk of nerve and blood vessel damage [13, 14]. Based on the above advantages, many surgeons have shown a great interest in CS fixation technology, which may be a replacement for traditional PS fixation.

Currently, the CS technique and the PS technique are both used in lumbar interbody fusion. However, which technology should be used as the gold standard is still controversial. Therefore, we conducted a systematic review and meta-analysis of existing clinical studies to establish the best evidence to resolve this controversy.

## Methods

### Literature search strategy

Systematic literature searches were performed in five electronic databases, including PubMed, Cochrane Library, MEDLINE, Web of Science, and EMBASE. We searched using the following combination of MeSH (Medical Subject Heading) terms and free text words: “cortical bone trajectory,” “cortical screw,” “pedicle screw,” and “lumbar spine.” The search date was from the time when databases were built on 18 March 2019. We did not restrict searches based on language or publication year. To prevent certain studies from being missed, we manually searched the bibliographies of randomized controlled trials (RCTs), meta-analyses, and systematic reviews.

### Selection of studies

The study inclusion and exclusion processes were divided into two groups. The selection was first based on the title and abstract, and if a decision could not be made from the summary, the full text would be retrieved. When there was a disagreement between the two groups, the selection committee would discuss until a consensus was reached.

### Inclusion and exclusion criteria

We have included studies that met the following criteria: (1) included studies were RCTs or comparative studies; (2) a comparative study on the efficacy of CS and PS techniques in lumbar interbody fusion; (3) average follow-up time of more than 1 year; and (4) the comparison outcomes included at least one of the following: surgical time, intraoperative blood loss, length of stay, back and leg pain VAS, Oswestry Disability Index (ODI), Japanese Orthopaedic Association (JOA) scale, complications, and fusion rate.

Studies were excluded according to the following criteria: (1) editorials, letters, reviews, case reports, and cadaver or animal experiments; (2) robotic-assisted fixation and percutaneous internal fixation; (3) the patient was diagnosed with scoliosis, infection, or tumor; and (4) the data of the comparison outcomes could not be extracted.

### Data extraction

Two reviewers used standardized data extraction tables for data extraction. The extracted data included authors, publication date, title, country, study design, follow-up duration, number of patients, mean age of patients, type of operation, and comparison outcomes. The comparison outcomes included surgical time, intraoperative blood loss, length of stay, back and leg pain VAS, ODI, JOA, complications, and fusion rate. All data were extracted from article texts, tables, and figures. If there was missing information, the research author would be contacted to obtain the missing data or further information. Two reviewers independently extracted the data; if there were differences, they were resolved through discussion, and a consensus was reached by including third parties. The data extraction outcomes are shown in Table 1.

**Table 1:** Characteristics of included studies

Author (years)	Country	Study type	Number of samples	Gender (male)	Average age	Follow-up (months)	Technique of fusion	Outcomes
			CS/PS	CS/PS	CS/PS	CS/PS	CS/PS	
Hoffman et al. (2019) [15]	USA	Cohort	23/35	16/16	48.5/53.4	52.5/52.5	MIDLF/TLIF	1, 2, 3, 4, 5, 6, 8, 9
Marengo et al. (2018) [16]	Italy	Cohort	20/20	12/9	45.75/54	12/12	PLIF/PLIF	1, 2, 3, 4, 6, 8, 9, 10
Lee and Ahn (2017) [17]	Korea	RCT	35/37	31/33	51.2/51.7	24/24	PLIF/PLIF	4, 5, 6, 9, 10
Takenaka et al. (2017) [18]	Japan	Cohort	42/77	18/31	65.8/66.0	17/35.4	PLIF/PLIF	1, 2, 4, 5, 8, 9, 10
Sakaura et al. (2017) [19]	Japan	Cohort	22/20	4/6	70.7/68.3	39/35	PLIF/PLIF	1, 2, 7, 8, 9, 10
Peng et al. (2017) [20]	China	Cohort	51/46	23/21	62.8/61.9	12/12	PLIF/PLIF	1, 2, 3, 6, 8, 9, 10
Sakaura et al. (2016) [21]	Japan	Cohort	95/82	46/36	68.7/67.0	39/35	PLIF/PLIF	1, 2, 7, 8, 9, 10
Orita et al. (2016) [22]	Japan	Cohort	20/20	11/12	63.5/63.7	12/12	TLIF/TLIF	1, 2
Hung et al. (2016) [14]	China	Cohort	16/16	6/5	60.37/63.12	18/18	PLIF/PLIF	1, 2, 3, 4, 5, 6, 7
Ninomiya et al. (2016) [23]	Japan	Cohort	11/10	7/5	62.2/61.4	12/12	PLIF/PLIF	9
Chin et al. (2016) [24]	USA	Cohort	30/30	18/15	48/62	24/24	NM/NM	1, 2, 4, 5, 6
Lee et al. (2015) [13]	Korea	RCT	38/39	33/34	51.3/51.9	12/12	PLIF/PLIF	1, 2, 3, 4, 5, 6, 9, 10

Outcomes: 1. Blood loss, 2. Operating time, 3. Length of stay, 4. Visual analog score (back pain), 5. Visual analog score(leg pain), 6. Oswestry Disability Index, 7. Japanese Orthopaedic Association, 8. Intraoperative complications, 9. Postoperative complications, 10. Fusion rate. *CS*: cortical screw *PS*: pedicle screw *RCT*: Randomized controlled trial *MIDLF*: midline lumbar fusion *TLIF*: transforaminal lumbar interbody fusion *PLIF*: posterior lumbar interbody fusion *NM*: not mentioned

### Data analysis

We used Review Manager Version 5.3 (Copenhagen: The Nordic Cochrane Centre, The Cochrane Collaboration) to analyze the data of all outcomes and compare the CS fixed group with the PS fixed group. For continuous outcomes, such as surgical time, intraoperative blood loss, length of stay, back and leg pain VAS, ODI, and JOA, the means and standard deviations were pooled to a weighted mean difference (WMD) and 95% confidence interval (CI). Risk ratios (RRs) and 95% CI were used to evaluate the dichotomous outcomes, such as complications and fusion rate. We used *I*^2^ to quantify heterogeneity. If *I*^2^ > 50%, the heterogeneity was significant and the unstandardized mean difference was estimated using a random effects model. Otherwise, a fixed effects model was applied.

### Quality assessment

For non-randomized controlled trials (N-RCTs), the modified Newcastle Ottawa Scale (NOS) was used to assess the risk of bias [25]. Three domains in NOS were evaluated, including selection, comparability, and exposure, totaling 9 points (Table 2). For RCTs, the Cochrane Handbook for Systematic Reviews of Interventions was used, which includes seven domains: random sequence generation, allocation concealment, blinding of participants and personnel, blinding of outcome assessment, incomplete outcome data, selective outcome reporting, and other sources of bias (Fig. 2). The quality assessment was carried out independently by two reviewers and discussed with a third party if there were any disagreements.

**Table 2:** Quality assessment of cohort studies according to the Newcastle Ottawa Scale (NOS)

Author	Selection	Comparability	Exposure	Total score
Hoffman et al.	2	2	3	7
Marengo et al.	3	2	3	8
Takenaka et al.	2	2	3	7
Sakaura et al.	3	2	3	8
Chin et al.	3	2	3	8
Sakaura et al.	3	2	3	8
Orita et al.	3	2	2	7
Hung et al.	3	2	3	8
Ninomiya et al.	3	2	2	7
Peng et al.	3	2	3	8

## Results

### Literature search

**Fig. 1:**
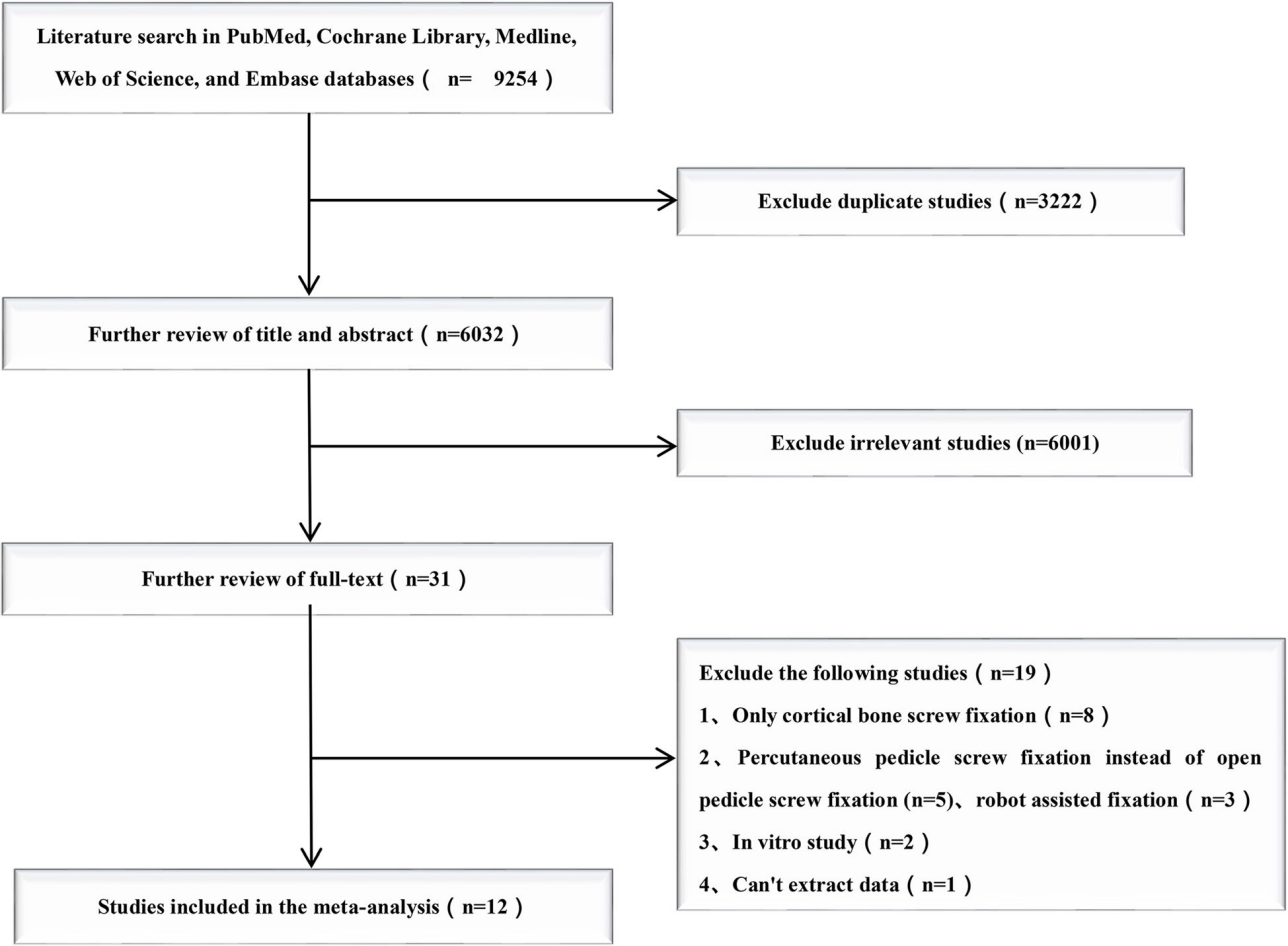
Flow diagram of the study selection

**Fig. 2:**
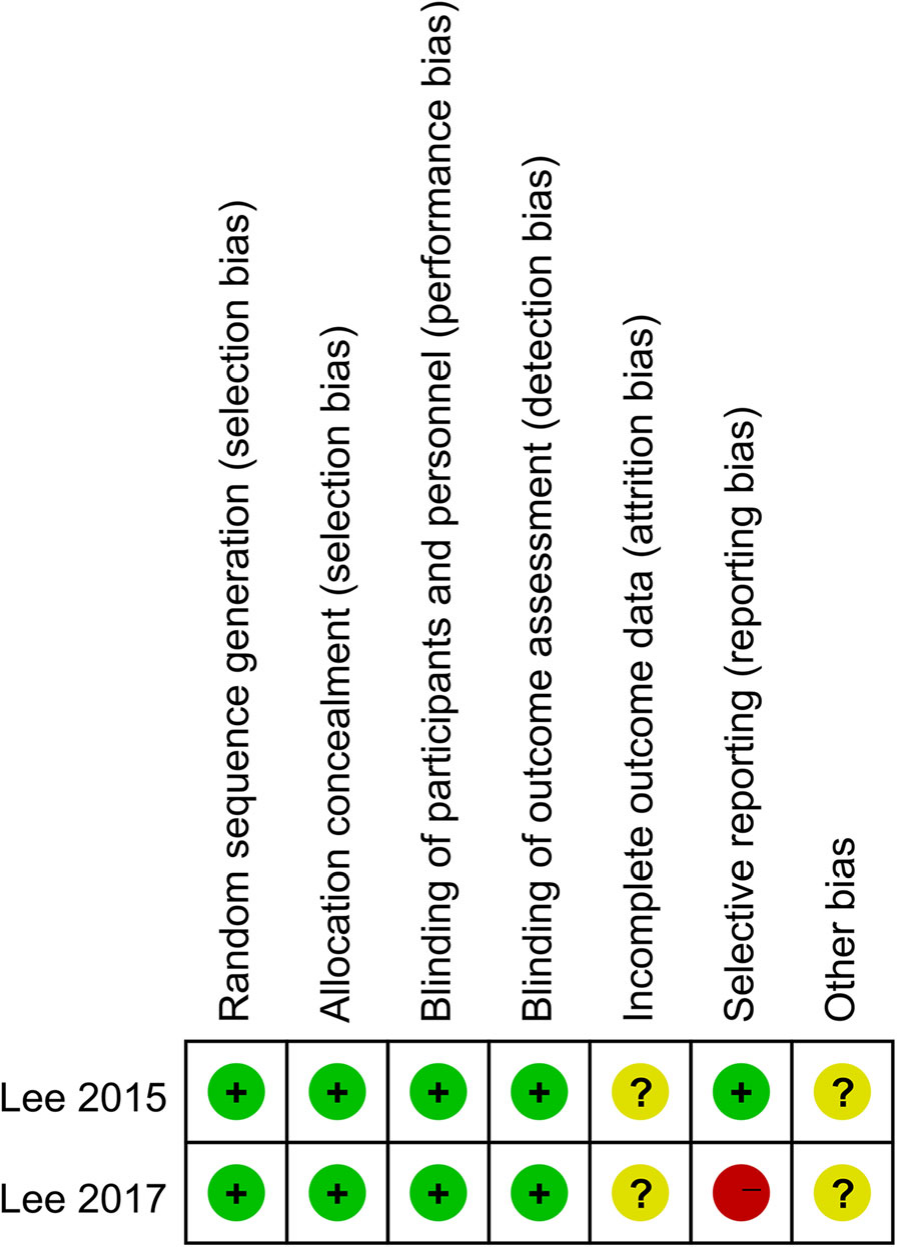
The methodological quality of the randomized controlled trials

There were 9254 studies identified from five electronic databases (Fig. 1). Of those, 3222 studies were duplicates, and 6001 studies were excluded after title and abstract screening. After careful full-text evaluation, 12 studies [13–24] were reviewed, and the data were extracted (Fig. 2). The demographic and clinical characteristics of the 12 studies are described in Table 1. There were 10 cohort studies [14–16, 18–24] and two RCTs [13, 17] included. A total of 403 patients who underwent CS fixation were compared with 432 patients who underwent conventional PS fixation. The mean follow-up time was more than 1 year, and the mean ages of the patients were 48–70 years old. Operating times and intraoperative blood loss were reported for 10 studies [13–16, 18–22, 24]. The length of stay was reported in five studies [13–16, 20]. Back and leg pain VAS was reported in seven studies [13–18, 24] and six studies [13–15, 17, 18, 24], respectively. ODI and JOA scores were reported in seven studies [13–17, 20, 24] and three studies [14, 19, 21], respectively. Complications and fusion rates were reported in nine studies [13, 15–21, 23] and seven studies [13, 16–21], respectively. Complications were divided into intraoperative and postoperative, including dural tear, screw dislocation, hematoma, wound infection, adjacent segmental disease (ASD), cage subsidence, and recurrent radiating pain in the lower extremities. The posterior lumbar interbody fusion (PLIF) procedure was performed in nine studies [13, 14, 16–21, 23]. The transforaminal lumbar interbody fusion (TLIF) procedure was performed in one study [22]. Two studies performed either a midline lumbar fusion (MDLF) or a TLIF in combination with the CS and PS procedures. One study [24] did not mention which fusion technology was performed. One study [17] was followed up for 2 years after another study [13]. To avoid repeated comparisons of the study patients, our meta-analysis included only partial outcomes from the previous study [13]: operating time, length of stay, and intraoperative blood loss.

### Intraoperative blood loss

**Fig. 3:**
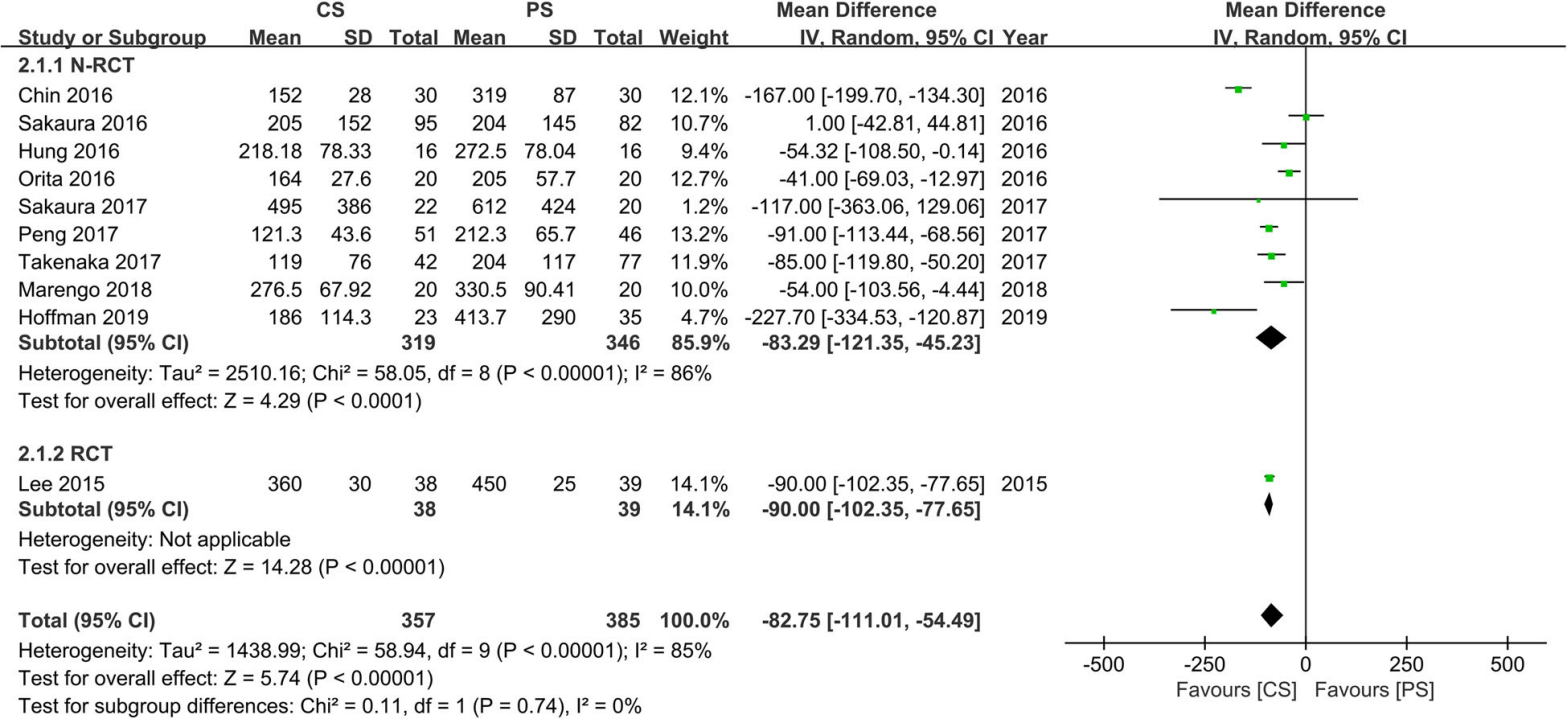
Meta-analysis of CS fixation group versus PS fixation group in intraoperative blood loss

Ten studies [13–16, 18–22, 24] with 357 and 385 patients, respectively, compared the mean intraoperative blood loss between the CS and PS groups. The pooled outcomes indicated that the CS group had significantly lesser intraoperative blood loss than the PS group (WMD, − 82.75; 95% CI, − 111.01 to − 54.49; *p* < 0.05). The heterogeneity test outcome (*I*^2^ = 85%) indicated significant heterogeneity. The outcomes showed that the use of CS fixation in lumbar interbody fusion can significantly reduce intraoperative blood loss compared with PS fixation (Fig. 3).

### Operating time

**Fig. 4:**
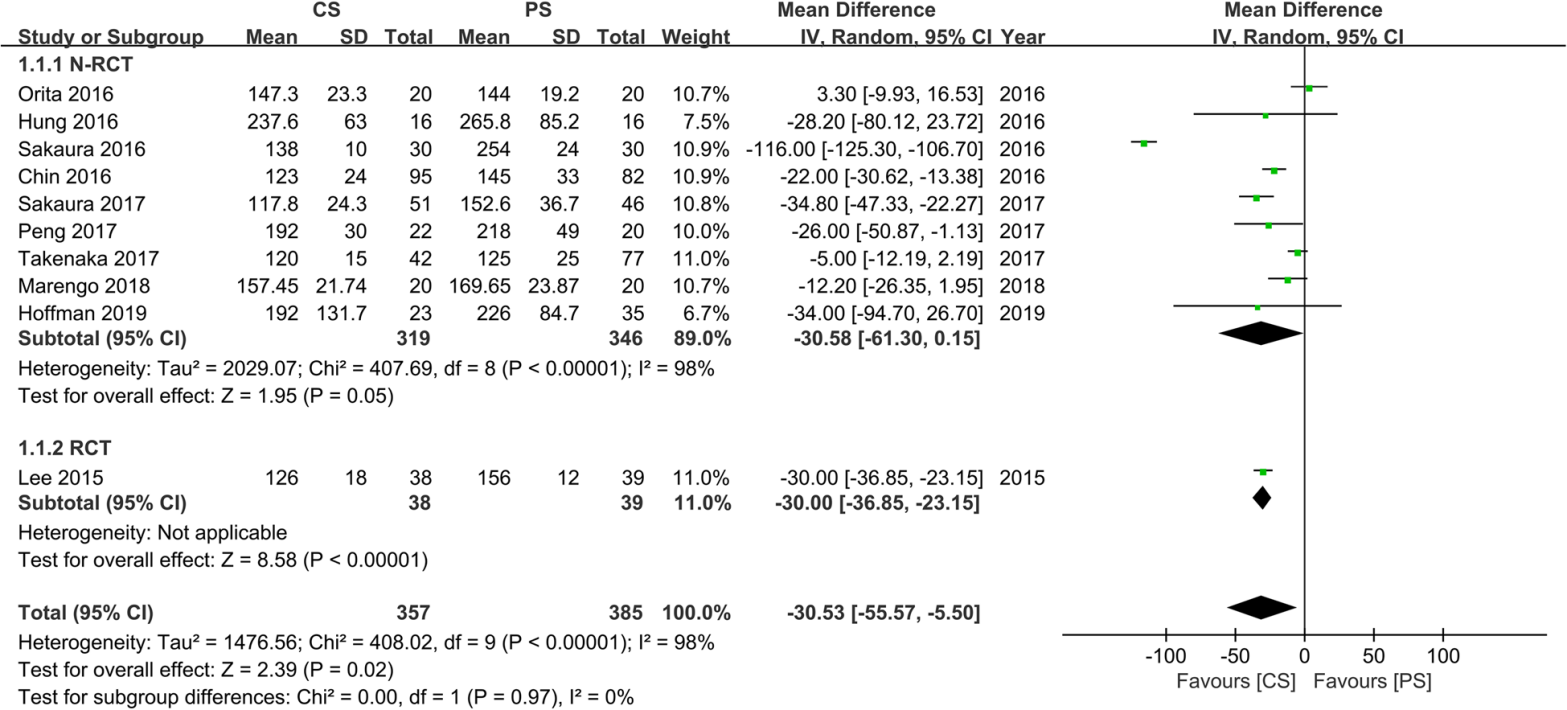
Meta-analysis of CS fixation group versus PS fixation group in operating time

Ten studies [13–16, 18–22, 24] with 357 and 385 patients, respectively, compared the mean operating time between the CS and PS groups. The pooled outcomes indicated that the CS group had significantly lesser operating times than the PS group (WMD, − 30.53; 95% CI, − 55.7 to − 5.50; *p* < 0.05). The heterogeneity test outcome (*I*^2^ = 98%) indicated significant heterogeneity. The outcomes indicated that the use of CS fixation in the lumbar interbody fusion can significantly reduce the operating time, compared with PS fixation (Fig. 4).

### Complications

**Fig. 5:**
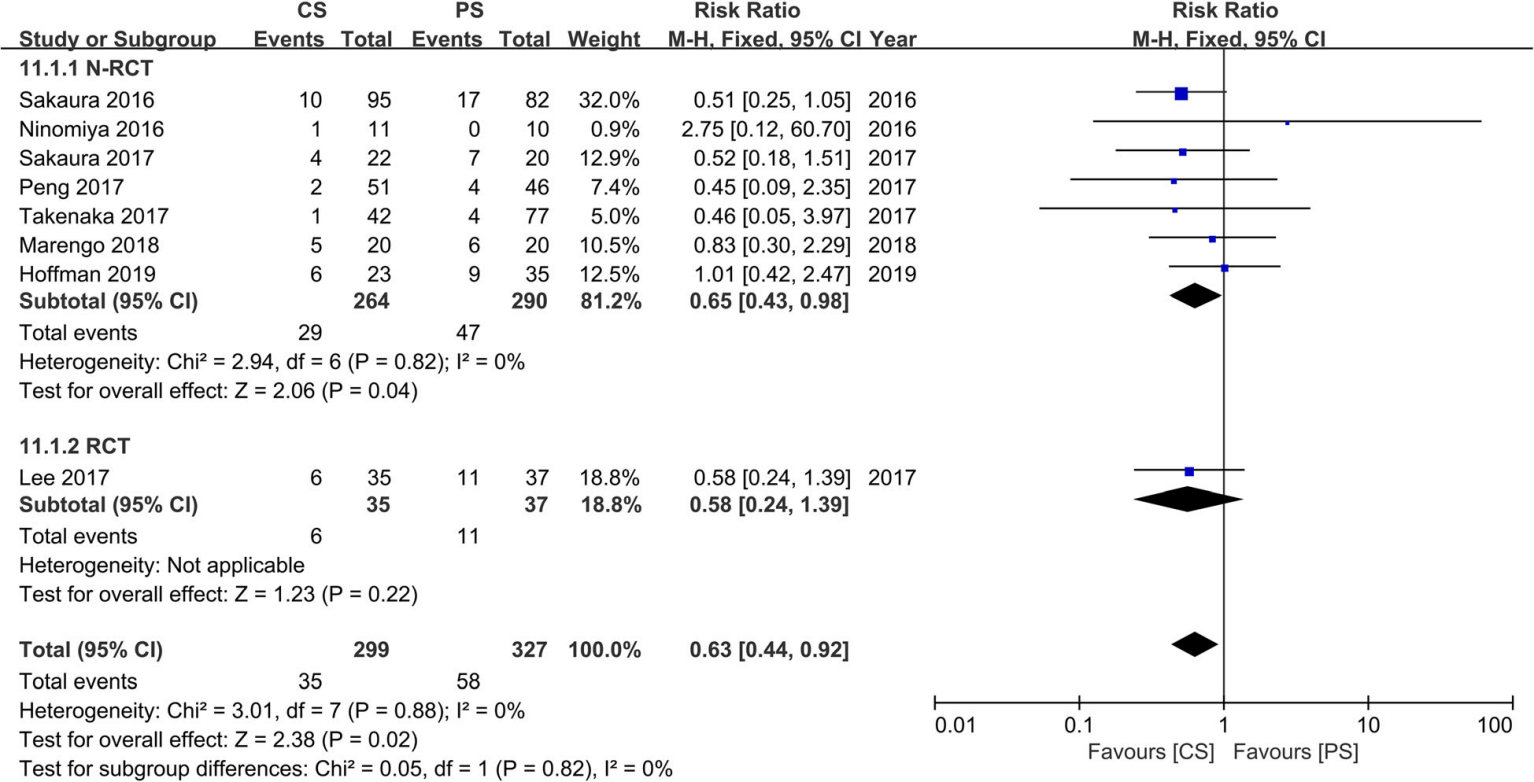
Meta-analysis of CS fixation group versus PS fixation group in the incidence of complications

Eight studies [15–21, 23] with 299 and 327 patients, respectively, compared complications between the CS and PS groups. We divided the N-RCT and RCT into two subgroups for the meta-analysis. In the N-RCT subgroup, the outcomes indicated that the incidence of complications in the CS group was significantly lower than that in the PS group (RR, 0.65; 95% CI, 0.43–0.98; *p* < 0.05). In the RCT subgroup, the meta-analysis indicated no significant differences between the CS group and the PS group (RR, 0.58; 95% CI, 0.24–1.39; *p* > 0.05). However, the pooled outcomes indicate that the CS group had a significantly decreased incidence of complications than the PS group (RR, 0.63; 95% CI, 0.44–0.92; *p* < 0.05). The outcome of the heterogeneity test was *I*^2^ = 0, and the fixed effects model was applied (Fig. 5).

**Fig. 6:**
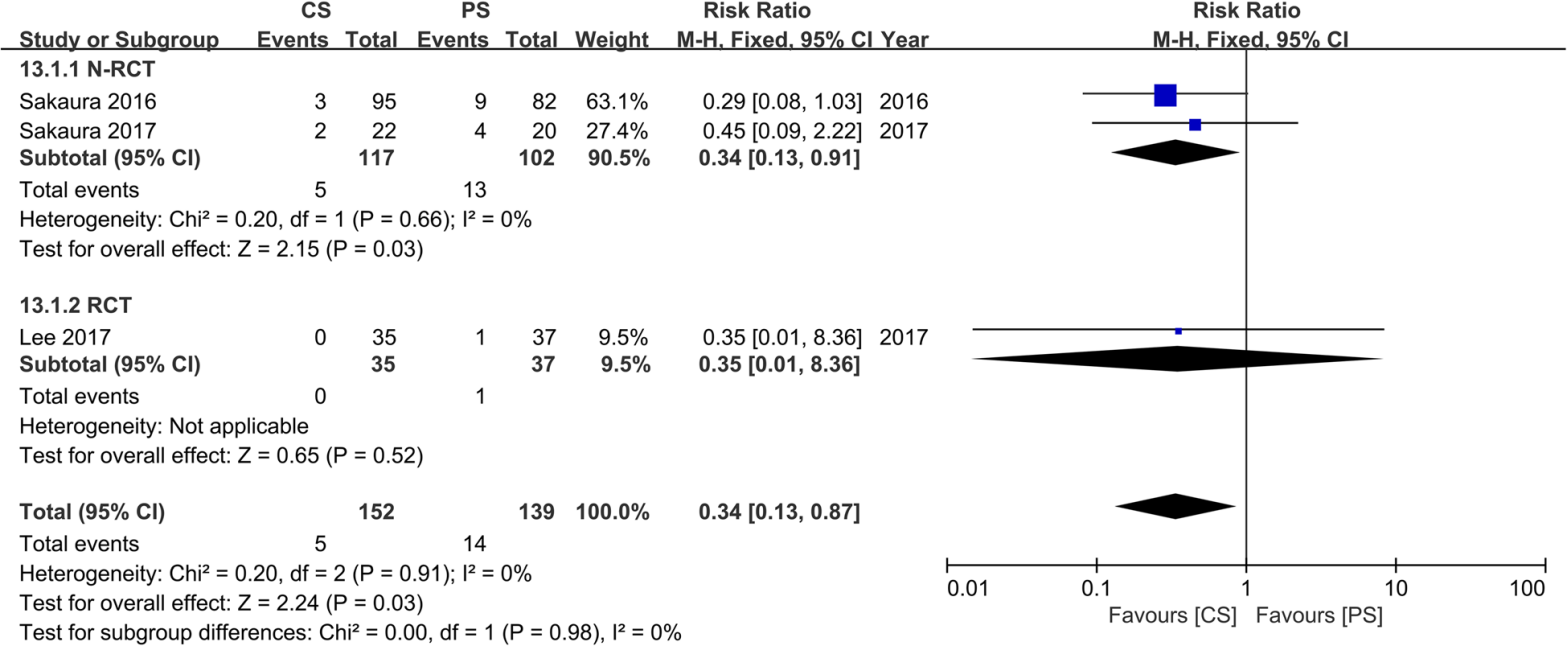
Meta-analysis of CS fixation group versus PS fixation group in the incidence of ASD

Three studies [17, 19, 21] with 152 and 139 patients, respectively, compared ASD between the CS and PS groups. The pooled outcomes indicated the CS group had a significantly lesser incidence of ASD than the PS group (RR, 0.34; 95% CI, 0.13–0.87; *p* < 0.05). The heterogeneity test outcome was *I*^2^ = 0, and the fixed effects model was applied (Fig. 6).

### Length of stay

**Fig. 7:**
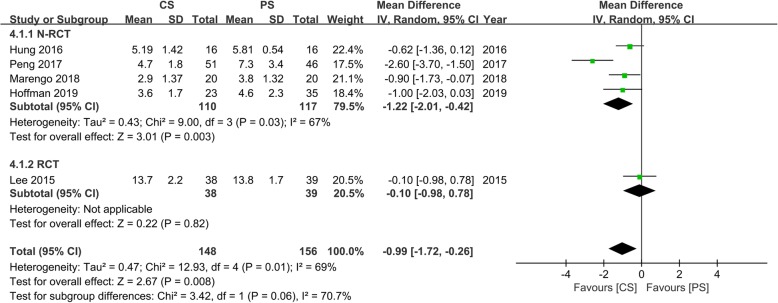
Meta-analysis of CS fixation group versus PS fixation group in the length of stay

Five studies [13–16, 20] with 148 and 156 patients, respectively, compared the mean lengths of stays between the CS and PS groups. The pooled outcomes indicated that the CS group had significantly shorter lengths of stays than the PS group (WMD, − 0.99; 95% CI, − 1.72 to − 0.26; *p* < 0.05). The heterogeneity test outcome (*I*^2^ = 69%) indicated a slightly higher heterogeneity. The outcomes of the meta-analysis indicated that the use of CS fixation in lumbar interbody fusion can significantly reduce the length of stay compared with PS fixation (Fig. 7).

### Back and leg pain VAS

**Fig. 8:**
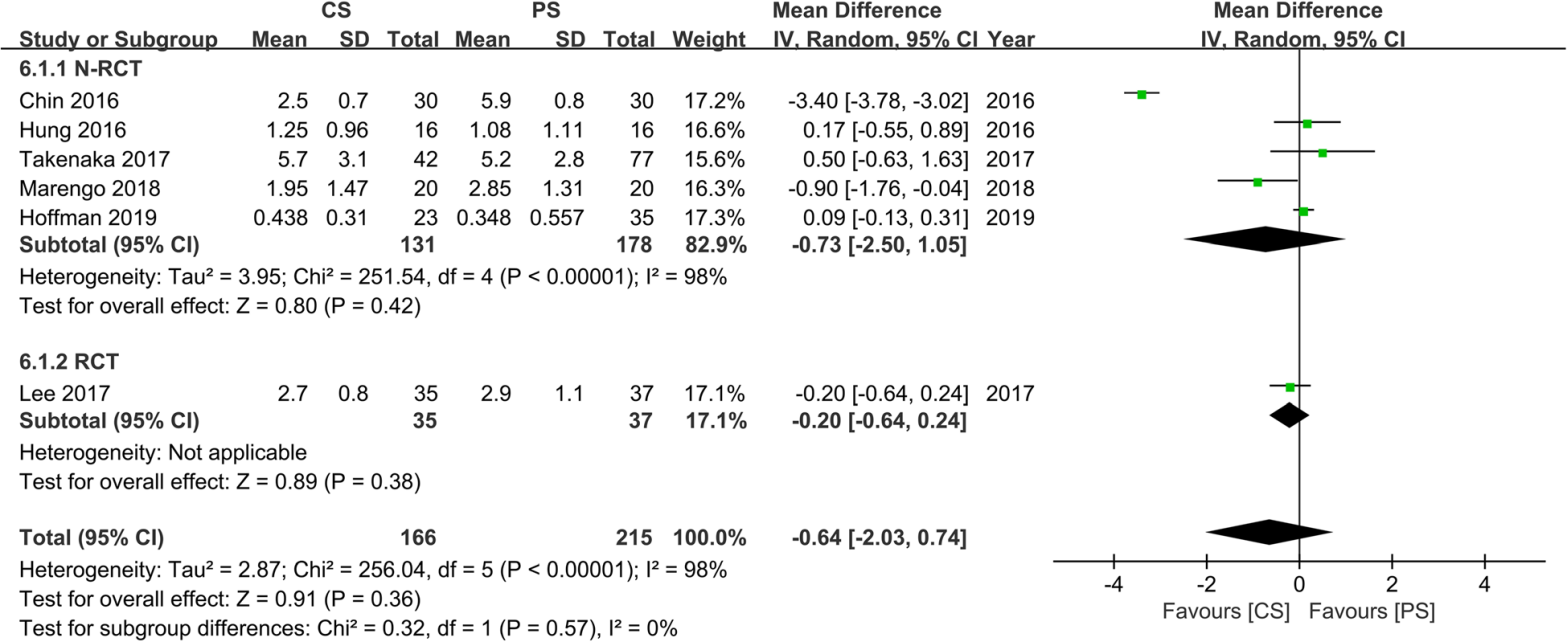
Meta-analysis of CS fixation group versus PS fixation group in back pain VAS

Six studies [14–18, 24] with 166 and 215 patients, respectively, compared the mean back pain VAS between the CS and PS groups. The meta-analysis indicated that there were no significant differences between the CS and PS groups (WMD, − 0.64; 95% CI, − 2.03–0.74; *p* > 0.05). The heterogeneity test outcome (*I*^2^ = 98%) indicated significant heterogeneity (Fig. 8).

**Fig. 9:**
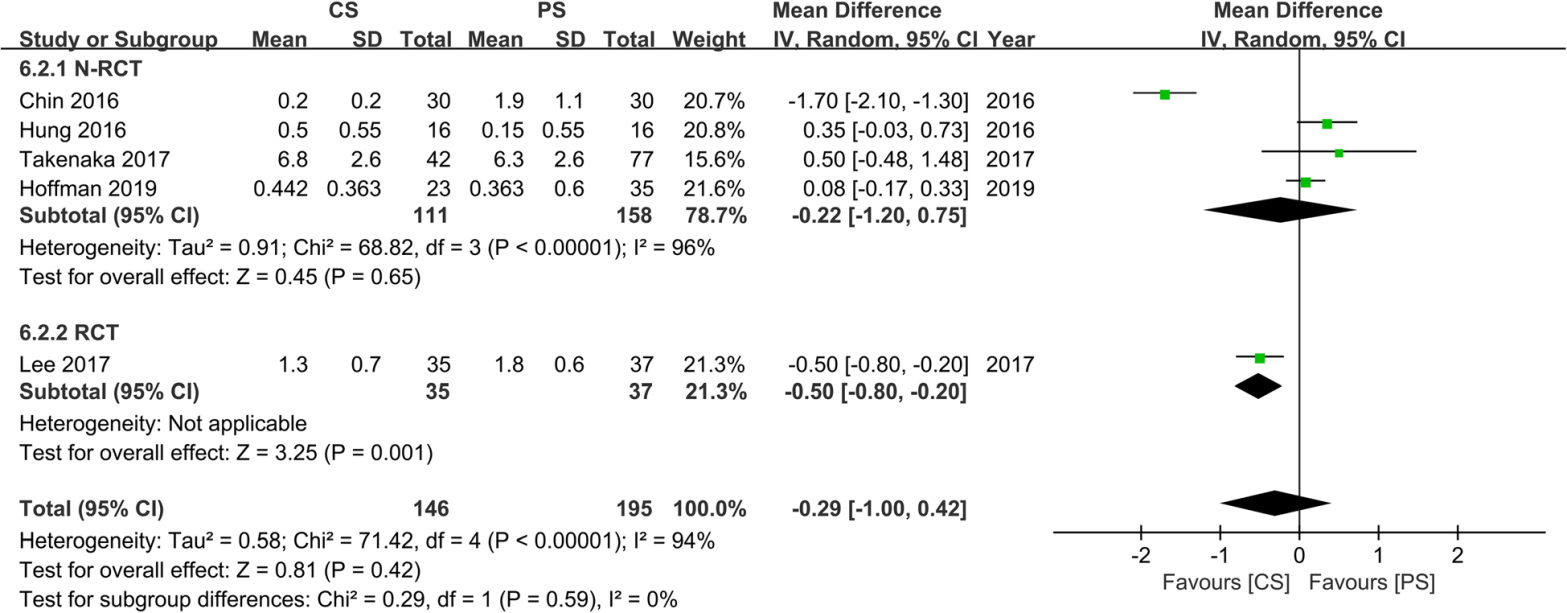
Meta-analysis of CS fixation group versus PS fixation group in leg pain VAS

Five studies [14, 15, 17, 18, 24] with 146 and 195 patients, respectively, compared the mean leg pain VAS between the CS and PS groups. We divided the N-RCT and RCT into two subgroups for meta-analysis. In the N-RCT subgroup, the meta-analysis indicated no significant differences between the CS group and the PS group (WMD, − 0.22; 95% CI, − 1.20–0.75; *p* > 0.05). In the N-RCT subgroup, the outcomes indicated that the leg pain VAS in the CS group was significantly lower than that in the PS group (WMD, − 0.50; 95% CI, − 0.80 to − 0.20; *p* < 0.05). However, the pooled outcomes indicated there was no significant difference between the CS and PS groups (WMD, − 0.29; 95% CI, − 1.00–0.42; *p* > 0.05). The heterogeneity test outcome (*I*^2^ = 94%) indicated significant heterogeneity (Fig. 9).

### ODI and JOA scores

**Fig. 10:**
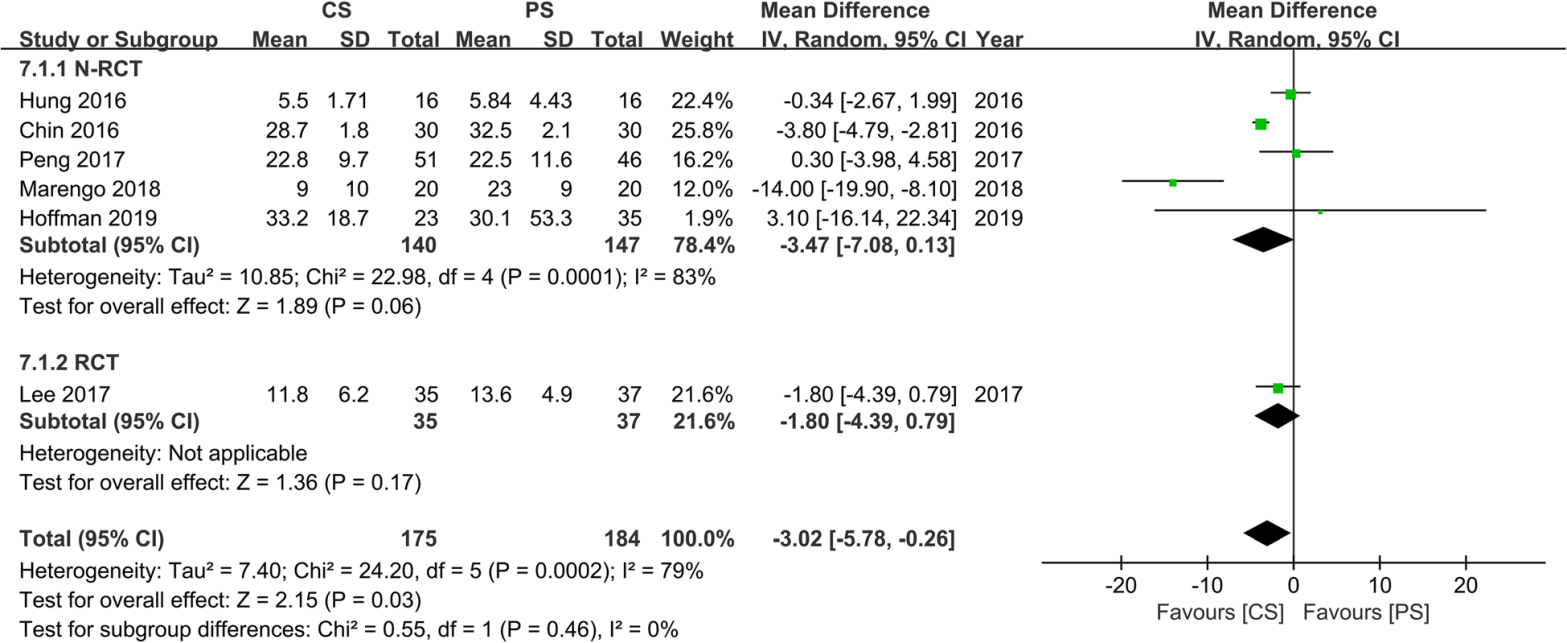
Meta-analysis of CS fixation group versus PS fixation group in ODI score

Six studies [14–17, 20, 24] with 175 and 184 patients, respectively, compared the mean ODI scores between the CS and PS groups. Meta-analysis indicated the ODI scores in the CS group was significantly lower than that in the PS group (WMD, − 3.02; 95% CI, − 5.87 to − 0.26; *p* < 0.05). The heterogeneity test outcome (*I*^2^ = 79%) indicated significant heterogeneity (Fig. 10).

**Fig. 11:**
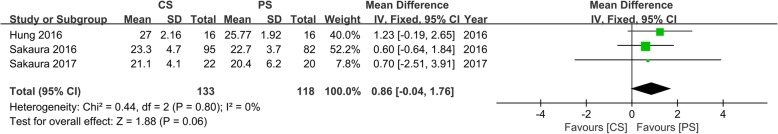
Meta-analysis of CS fixation group versus PS fixation group in JOA score

Three studies [14, 19, 21] with 133 and 118 patients, respectively, compared mean JOA scores between the CS and PS groups. The meta-analysis indicated no significant differences between the CS group and the PS group (WMD, 0.86; 95% CI, − 0.04–1.76; *p* > 0.05). The heterogeneity test outcome was *I*^2^ = 0, and the fixed effects model was applied (Fig. 11).

### Fusion rates

**Fig. 12:**
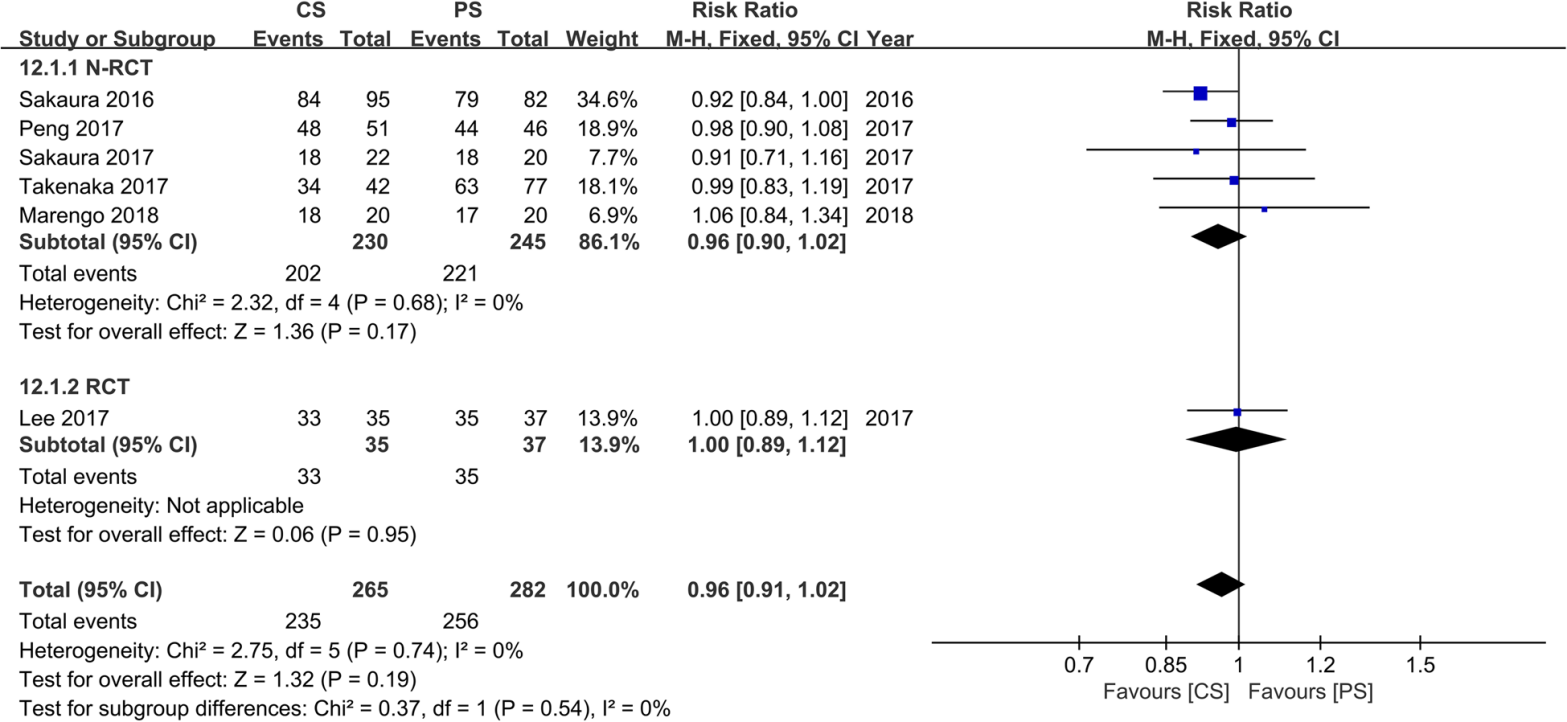
Meta-analysis of CS fixation group versus PS fixation group in fusion rates

Six studies [16–21] with 265 and 282 patients, respectively, compared fusion rates between the CS and PS groups. The meta-analysis indicated no significant differences between the CS group and the PS group (RR, 0.96; 95% CI, 0.91–1.02; *p* > 0.05). Heterogeneity test outcomes were *I*^2^ = 0, and the fixed effects model was applied (Fig. 12).

## Discussion

Lumbar fusion is a common surgical procedure for the treatment of various spinal diseases, and the frequency of such surgeries has increased significantly over the past 20 years [26]. At present, lumbar interbody fusion is an effective surgical procedure for the treatment of degenerative lumbar disease [27]. However, in lumbar interbody fusion, due to the use of PS internal fixation, several adverse outcomes can result, such as long surgical incision, excessive intraoperative blood loss, excessive muscle soft tissue damage, and superior facet joint violations [8, 28, 29]. Therefore, the CBT screw fixation technology was proposed to make up for these drawbacks [9]. Compared with traditional PS fixation techniques, CS fixation technology increases pull-out strength, reduces intraoperative paraspinal muscle stripping, and reduces the risk of superior facet joint violations [10, 14, 16]. Several clinical and imaging studies evaluated CBT screw placement, and the outcomes indicated that CBT has the following advantages: high safety, easy placement, and good cortical bone contact [30, 31]. Some studies even have indicated that CS fixation in single-segment lumbar interbody fusion can replace PS fixation [17]. Currently, there are only a few large-scale studies and related meta-analyses comparing the clinical outcomes of CS and PS fixation techniques in lumbar interbody fusion. Therefore, this meta-analysis is necessary. In our meta-analysis, the information of 835 patients was extracted from 12 published studies, including two RCTs and 10 cohort studies, respectively, using the Cochrane Handbook for Systematic Reviews of Interventions and NOS for quality assessment. The outcomes indicated that the included literature was of high quality. Our study indicated that the operating time, intraoperative blood loss, length of stay, incidence of complications, and ODI scores of CS internal fixation in lumbar interbody fusion were significantly lower than those in PS internal fixation. For the back and leg pain VAS, JOA scores, and intervertebral fusion rate, the meta-analysis outcomes indicated no significant differences between the CS group and the PS group.

Regarding the operating time, Dabbous et al. [32] indicated that the CS fixation technique was used to reduce the operation time. Chin et al. [24] indicated that the use of CS fixation during lumbar interbody fusion significantly reduced operating time compared with PS fixation. However, Hoffman et al. [15] indicated that there are no differences in operating time between CS fixation and PS fixation. Our outcomes indicated that the use of CS fixation can significantly reduce the operating time compared with PS fixation, which is likely due to differences in the surgeon’s surgical techniques and the patient’s body mass index (BMI). Studies [15, 33] have shown that the application of CS fixation in lumbar interbody fusion can significantly reduce intraoperative blood loss compared with PS fixation, which was consistent with our results. The reduction in intraoperative blood loss may be because traditional PS placement requires more tissue exposure and muscle damage than CS [14, 16]. Excessive muscle damage can also lead to a prolonged length of stay [34]. Marengo et al. [16] indicated that both CS-PLIF and PS-PLIF improved patient clinical outcome scores (back and leg pain VAS and ODI scores), and CS-PLIF improved more significantly. Keorochana et al. [35] indicated that the back and leg pain VAS using the CS fixation technique group was significantly better than the PS group, but there were no significant differences between the CS group and the PS group. Our meta-analysis showed similar outcomes. For the ODI score, Keorochana et al. [35] indicated that there was no difference between the CS and PS groups, but our study indicated that the CS group had a significantly better ODI score than the PS group. Marengo et al. [16] also obtained a similar outcome. For the fusion rate, the previous meta-analysis [16] yielded an outcome consistent with ours. In lumbar interbody fusion, CS fixation and PS fixation can achieve a good fusion effect, and there is no statistically significant difference between the two groups. For complications, Keorochana et al. [35] indicated that the incidence of surgical complications in the CS group was slightly lower than that in the PS group, but this decrease was not statistically significant. However, our meta-analysis yielded different outcomes: the incidence of surgical complications in the CS group was significantly lower than that in the PS group. Sakaura et al. [19] indicated that the incidence of ASD in the PS group was two times higher than in the CS group. Our meta-analysis also indicated that the incidence of ASD in the CS group was significantly higher than that in the PS group. The invasion rate of the facet joints was higher when the PS was placed, which further increased the biomechanical stress, resulting in the instability of adjacent segments [36, 37]. However, there is still controversy about whether CS fixation can lead to the occurrence of ASD.

The limitations of this meta-analysis include the following: only two RCTs were included, and most studies were non-randomized controlled trials and small sample designs, which are more prone to various types of bias; the outcomes of the study may be affected by different follow-up times; only one or two lumbar interbody fusion studies were included, which did not represent the long-segment fixation effect; and the heterogeneity of some of the study outcomes was significant, and this issue was not resolved by subgroup analyses of the RCTs and N-RCTs.

## Conclusion

Operating time, intraoperative blood loss, length of stay, incidence of complications, ASD incidence, and ODI score for the CS fixation technique are superior to those of the PS fixation technique. However, for back and leg pain VAS, JOA score, and intervertebral fusion rate, CS fixation and PS fixation achieved similar clinical outcomes. Based on current evidence, we suggest that CS fixation techniques can be used to replace PS fixation techniques in short-segment lumbar fusion. In the future, the CS fixation technique and PS fixation technique should be compared when used in long-segment lumbar interbody fusion and with regard to the incidence of ASD.

## Data Availability

All data generated or analyzed during this study are included in this published article and its supplementary information files.

## Abbreviations

ASD: Adjacent segmental disease
CBT: Cortical bone trajectory
CI: Confidence interval
CS: Cortical screw
JOA: Japanese Orthopaedic Association
MDLF: Midline lumbar fusion
NOS: Newcastle Ottawa Scale
ODI: Oswestry Disability Index
PLIF: Posterior lumbar interbody fusion
PS: Pedicle screw
RCTs: Randomized controlled trials
RRs: Risk ratios
TLIF: Transforaminal lumbar interbody fusion
VAS: Visual analog scale
WMD: Weighted mean difference
